# 2019-nCoV: Measures Adopted at the Departments of Oral Surgery and Radiology during the Period of an Uncontrolled Transmission Increase

**DOI:** 10.3390/dj8020057

**Published:** 2020-06-08

**Authors:** Lauren Bohner, Melanie Maus, Johannes Kleinheinz, Marcel Hanisch

**Affiliations:** Department of Cranio-Maxillofacial Surgery, University Hospital Münster, Albert-Schweitzer-Campus 1, Building W 30, D-48149 Münster, Germany; melanie.maus@ukmuenster.de (M.M.); johannes.kleinheinz@ukmuenster.de (J.K.); marcel.hanisch@ukmuenster.de (M.H.)

**Keywords:** Coronavirus infections, dental care, infection control

## Abstract

A new mutation of 2019-nCoV emerged and has been spreading worldwide. Dental practices are an important person-to-person transmission route. In this regard, preventive measures are required to avoid the cross contamination among professionals and patients. This report brings recommended measures for dental assistance during the pandemic phase. The clinical protocol applied at the Department of Oral Maxillofacial and Surgery, such as at the Department of Radiology, Hospital University Münster, is described. A management protocol was applied to prevent the transmission route of 2019-nCoV. Patients infected with 2019-nCoV are treated only in emergency situations. The use of protective equipment and dental office isolation were the major points to avoid the contact between infected and non-infected patients. Preventive measures should be taken in order to reduce the spread of 2019-nCoV infection.

## 1. Introduction

Since the end of 2019, a pneumonia infection has been emerging and widely spreading around the world. The novel mutation of coronavirus, known as 2019-nCoV, has reached more than 693,000 cases, resulting in approximately 33,100 deaths [1]. The mechanism of 2019-nCoV consists on the binding between angiotensin-converting enzyme 2 (ACE2) and SARS-CoV-2. This interaction can damage the alveolar cells of the lung, resulting in systemic complications, which in most severe cases may lead to death [2].

Clinical symptoms are fever, cough, fatigue, muscle pain and dyspnea. Headaches, hemoptysis and diarrhea can also be present [3,4]. As respiratory syndrome, anemia, heart injuries and secondary infections may occur, the treatment consists on mechanical ventilation, antibiotics, antiviral therapy and corticosteroids [5]. To date, no established protocol is available. Thus, preventive measures, such as the use of protection equipment, personal hygiene and ventilated rooms, are essential to avoid contamination [2].

The disease transmission occurs through direct or indirect contact with infected persons, regardless of the presence of clinical symptoms. Thus, not only patients presenting clinical symptoms, but also those asymptomatic or in the incubation period can be a source of transmission [6]. Considering that dental assistance can be an important transmission route, dental clinicians should be aware of preventive managements to protect professionals and patients [6,7]. The purpose of this report is to describe the protocol adopted by the Departments of Oral Surgery and Radiology, Hospital University Münster, to avoid the cross infection of professionals and patients during the phase of an uncontrolled transmission increase.

## 2. Report

### 2.1. General Recommendations for Dental Practices

While the disease control is not yet possible, elective treatments should be avoided. Emergency treatments of infected patients should be performed, when possible, in an isolated dental office. Thus, the first step is to identify the infected patient. Before the consultation, a questionnaire about acute clinical symptoms should be included in the medical history. Additionally, information regarding travels performed on the last 14 days, as well as social contact with infected persons, should be investigated. The measurement of body temperature is also essential to identify a febrile phase [6].

If the patient responds “no” to all questions and does not present fever, conventional treatment protocol might be applied. By responding “yes” to one of these questions but in the absence of fever, it is recommended to perform the treatment 14 days after the related event. In cases on which the patient´s body temperature is higher as 37.3 °C, a quarantine period is required [6].

Hand disinfection measures should be applied before and after each dental treatment. Furthermore, direct hand touch on eyes, mouth and nose should be avoided. Usual measures, such as use of personal protective equipment (PPE) and disinfection protocol after each patient consultation, should be respected. PPE includes the use of gloves, masks, eyewear, caps and coats. When treating infected patients, the use of a special mask, FFP-2 or FFP-3, is strongly recommended [7,8,9].

### 2.2. Current Measures for the Period of Uncontrolled Increase of COVID-19 Adopted at the Department of Oral and Maxillofacial Surgery, Hospital University Münster

The Department of Oral and Maxillofacial Surgery, Hospital University Münster, comprises maxillofacial physicians and dentists specialized in oral surgery. A protocol was adopted in order to ensure the health of clinicians, assistants, and patients during the pandemic phase. A Classification provided by the Occupational Health Service has been used to determine the risks to which professionals were exposed, as well as the required measures to avoid infection (Table 1).

The first step was to control the entrance of patients in the clinic. During the uncontrolled COVID-19 spread, only patients with a scheduled consultation are allowed to enter the dental clinics. Furthermore, the entrance of accompanying persons is not permitted.

At the moment of the outbreak, elective treatments were suspended for all patients, regardless the diagnosis with 2019-nCoV (Table 2). Non-elective treatments, as acute pain, abscess and head and neck tumors operation, are being performed as usually. Further conditions, as temporomandibular disorders or oral mucosal diseases, are analyzed according to the clinical situation. For instance, acute pain caused by a temporomandibular disorder, such as lesions that remain more than 14 days in oral cavity, are considered emergencies.

After controlling the acute phase, elective treatments are postponed to the end of the uncontrolled situation. The proper use of PPE is required throughout treatment, as well as during the room disinfection.

All patients arriving at the clinic are screened based on a COVID-19 questionnaire covering the items described above (See Section 2.1). Likewise, patient´s body temperature is checked with a non-contact thermometer. Patients are considered of high risk when reporting fever, cough, breathing complications, personal contact with a 2019-nCoV patient or travel history on the last two weeks. In case of a positive response, the treatment need is analyzed and, if required, a COVID test is taken. Conversely, when all questions are responded with NO and the body´s temperature is lower than 37.3 °C, the patient is considered of low-risk.

Prior to the treatment, the patient is advised to rinse with an alcohol-based mouthwash. For patients who do not tolerate alcohol, a chlorhexidin 0.12% mouthrinse is given. Treatments generating aerosols are avoided, being performed only under slightly conditions. For instance, an abscessed tooth is treated with incision and pharmacological therapy rather than tooth trepanation.

Likewise, for 2019-nCoV-patients at the acute phase, only non-elective treatments are performed. In order to avoid contact with patients who are not infected by the virus, 2019-nCoV-patients are treated in a separated dental office, away from both waiting room and conventional dental offices. During the patient management, dentists and assistants use all PPEs, including N95-masks (minimal FFP2) and disposable coats. As soon as the patient arrives the clinic, the department is advised to prepare the room. The patient is taken to the clinic at the time of the consultation, reducing exposure risks in the waiting room. On the entrance door there is a closet where the patient receive a disposable coat, gloves and a regular mask. The patient only removes the mask during the treatment. After the dental procedure, assistants wearing personal protective equipment decontaminate the room with an alcohol-based disinfection solution (Incidin plus 0.5%). Also, the room is not used in the next hour, if possible, to ensure the complete room disinfection.

### 2.3. Current Measures for the Period of an Uncontrolled Increase of COVID-19 Adopted at the Department of Radiology, Hospital University Münster

Control measurements are applied for all cases on which a radiograph is required. When possible, panoramic radiographs are preferred in order to prevent stimulate salivary flow caused by intra-oral radiographs. A dental assistant accompanies the patient to the Radiology center. During the exam, a radiology technician uses the same protective equipment described above. In order to ensure the absence of contamination, patient´s gloves are changed as soon as the patient enters to the Radiology Center.

As usual, the patient receives a protective gown and the radiograph exam is taken in accordance with a new protocol. In many cases, patient can continue to use the mask during the exam. Frankfurt horizontal plane and mid-sagittal plane are ensured by the patient positioning device. However, the use of a bite-block is avoided to prevent intra-oral manipulation. Instead, the patient is requested to bite on the anterior border of the tongue and, subsequently, to move the jaw anteriorly. This protrusion position simulates the bite conventionally taken on the bite-block.

Radiographic exams are taken from 2019-nCoV patients in a separated room, away from further radiological devices. This measurement is intended to avoid the contact of the patient with those who are not infected.

## 3. Conclusions

Preventive measures had to be taken to prevent the spread of 2019-nCoV infection. A special protocol was adopted to attend COVID and non-COVID patients during the phase of an uncontrolled disease control. However, measures should be modified according to each social and pandemic local situation.

- In summary, best practices should be improved during the uncontrolled disease increase. All patients should be considered to be infected, and some aspects must be taken into consideration during the dental treatment:
- Patient´s screening, as the avoidance of contact between COVID and non-COVID patients, are important measures to prevent spread of the disease;
- The correct use of PPE should be reinforced. When possible, a FFP-mask (minimal FFP2) should be used;
- A special attention must be given for the use of PPE and disinfection protocols. Hand hygiene and room disinfection must be performed between dental treatments;
- Aerosol generation should be avoided and conservative treatments should be preferred.

## Figures and Tables

**Table 1:** Risk assessment for professionals (Occupational Health Service, Hospital University Münster).

Status	No Contact with Infected Person	Contact with Infected Person	Permanence in Regions with COVID-19 Cases
No symptoms	No measurements	Quarantine (14 days)	No measurements
Clinical symptoms	To avoid contact Consultation with the physician	Consultation with the physician Quarantine (14 days)	To avoid contact Consultation with the physician

**Table 2:** Elective and non-elective treatments performed at the Department of Oral and Maxillofacial Surgery, Hospital University Münster.

Non-Elective Treatments	Elective Treatment
Abscess, acute infection	Dentoalveolar surgery
Hemorragie	Third molars Exraction
Tracheotomie	Implantology
Maxillofacial and dental trauma	Cystectomy
Tumor	Sinus treatment
Osteonecrosis	Orthognathic surgery
